# Book Review: Gas Biology Research in Clinical Practice, edited by Toshikazu Yoshikawa and Yuji Naito

**DOI:** 10.1186/2045-9912-1-25

**Published:** 2011-10-03

**Authors:** Atsunori Nakao, Yoshiya Toyoda

**Affiliations:** 1 Departments of Surgery and Cardiothoracic Surgery, University of Pittsburgh Medical Center, Pittsburgh, Pennsylvania, USA

## 

Gaseous substances constitute a unique class of indispensable biomaterials for maintaining the homeostasis of biological systems. In particular, the gases, including nitric oxide (NO), carbon monoxide (CO), hydrogen sulfide (H_2_S), and hydrogen (H_2_), have well been described in the last decade as signaling gas molecules, as well as other important candidates for clinical applications. Given the fact that those individual gases play critical roles in biological systems, providing the newest findings and applications for gas biology basic and clinical research is of great importance. This book provides valuable information not only for basic researchers in physiology and biochemistry, but also for clinicians who wish to learn more about the role of gaseous mediators. This book has been published with 32 figures and 11 tables and divided into a preface and seven sections, listed below. In each section, leading specialists share their scientific experience in the field, covering a wide range of topics, including genetic, physiological, and medical imaging techniques. In each chapter, the authors take the reader on a vast journey through the impressive recent advances in gas biology (Figure 1).

**Figure 1:**
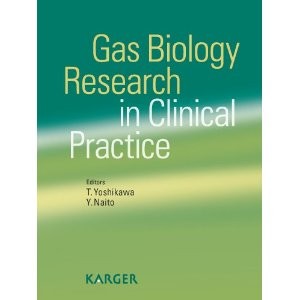
**Cover of "Gas Biology Research in Clinical Practice" edited by Toshikazu Yoshikawa and Yuji Naito**. (Published by Karger, Basel, Switzerland, 2011, ISBN 978-3-8055-9664-0).

The preface provides the background and acknowledgements. Drs. Yoshikawa and Naito have held annual meetings entitled "Heme Oxygenase Research Forums" since 2004. These conferences largely expanded and encouraged CO research, which was generated in the process of heme degradation by heme oxygenase. The idea of organizing this type of book was initiated by Dr. Hideo Ueda, who has been ill for years. As the subject matter of this book is of importance for gas biology research, Drs. Yoshikawa and Naito took over and completed Dr. Hideo's work.

The first "General" section is composed of three classic review papers which provide the necessary introduction to gas biology. The first chapter, entitled "Roles of Stress-Inducible Carbon Monoxide in the Regulation of Liver Function," was written by Dr. Makoto Suematsu, *et al. *(pages 1-5). As the liver is a gigantic resource of CO derived from heme degradation *in vivo*, constitutive and inducible CO has been suggested to regulate porto-sinusoidal vascular and biliary function. Interestingly, Dr. Suematsu mentioned that cystathionine β-synthase (CBS), a heme-containing enzyme, acts as a CO sensor. The enzyme CBS is also known to be an important synthesizer of H_2_S; therefore this chapter suggests cross-communication among the biological signaling gas system. Dr. Yoshihisa Urita *et al. *cover "Intraluminal Gas and Gastrointestinal Disease" (pages 6-14). Gases are produced while passing through the gastrointestinal tract, possibly causing abdominal symptoms, while gas is continuously removed by eructation, anal evacuation, absorption through the intestinal mucosa, and bacterial consumption. Impaired gas movement in the alimentary tract is more closely associated with abdominal symptoms than is liquid movement. Intestinal gas is often cited as a major cause of irritable bowel syndrome (IBS). The last chapter of this section covers "Therapeutic Medical Gas" (pages 15-23), providing background and clinical feasibility of various therapeutic medical gases, such as NO, CO, H_2_S, H_2_, xenon, helium, and ozone. This chapter includes a table that lists potential therapeutic medical gases and summarizes their chemistry or biological effects (page 16).

Section two, entitled "Gas and Medical Application: I. CO," starts with the chapter "Analysis of Breath CO and Application to Hemodynamic Monitoring," which describes monitoring methods using exhaled CO and its physiological roles on alimentary tracts. Dr. Sawano developed accurate, non-invasive, continuous carboxyhemoglobin densitometry by expired gas analysis (pages 24-34). Application of this technique to low-dose carboxyhemoglobin dilution achieved minimally invasive estimation of cardiac output and circulating blood volume. In the second chapter, Dr. Naito *et al. *summarize "CO and Its Application to Gastrointestinal Disease" (pages 35 to 42). Endogenous and exogenous CO have been reported to have anti-inflammatory and anti-apoptotic effects by various mechanisms and are involved in attenuating colonic mucosal inflammation. Considering the fact that cigarette smoking protects against the development of ulcerative colitis, the authors assume that CO, one of the components of cigarette smoke, might play an important role in ameliorating colonic inflammation.

Section three, "Gas and Medical Application: II. NO," consists of two chapters written by NO research expert groups Dr. Maruyama *et al. *and Dr. Shime *et al. *This section focuses on the mechanisms of inhaled NO's action and clinical applications, particularly focusing on persistent pulmonary hypertension of the newborn, pulmonary hypertension after cardiac surgery, and acute lung injury/acute respiratory distress syndrome (ALI/ARDS). A meta-analysis of 12 randomized controlled trials does not indicate the routine use of inhaled NO in patients with ARDS, although it is currently performed as a rescue therapy in patients with severe ARDS symptoms who require extracorporeal membrane oxygenation. In addition, other clinical indications of inhaled NO, such as chronic obstructive pulmonary disease (COPD), heart transplantation, and congestive heart failure, are listed and discussed.

Like NO and CO, H_2_S has been recognized not only as a pollutant, but also as an important gaseous physiological mediator. Section four, entitled "Gas and Medical Application: III. H_2_S," covers the basic/clinical aspects of H_2_S regarding the alimentary tract. Dr. Chan Young Ock *et al. *discuss "Hydrogen Sulfide in the Gastrointestinal Tract: Friend or Foe?" (pages 65-72). They propose H_2_S's great pharmacological potential in the gastrointestinal tract. However, they concluded that H_2_S may be a double-edged sword in the gastrointestinal tract; it acts an anti-inflammatory mediator to inhibit leukocyte activation, but when overproduced, may contribute to further inflammation in an already inflamed area. Dr. Tomohisa Takagi *et al. *discuss "Role of Hydrogen Sulfide in Colitis" (pages 73-80). They demonstrate that H_2_S has been implicated in the regulation of intestinal inflammation. Dr Takeuchi *et al. *focus on the duodenum and report their work in the chapter entitled "HCO_3_^- ^Stimulatory Action of Hydrogen Sulfide in rat Duodenum" (pages 81-90). Using a rat duodenum loop between the pyloric ring and the proximal side of the outlet of a common bile duct, they showed that NaHS (H_2_S donor) increased duodenal HCO_3_^- ^secretion via the mechanism mediated by prostaglandin E_2 _and NO.

Clinical application of hydrogen gas was discussed in section five, entitled "Gas and Medical Application: IV. H_2_" (pages 91-99). Hydrogen is one promising gaseous agent that has come to forefront of research during the last few years. A number of basic and clinical researchers have revealed that hydrogen is an important physiological regulatory factor with antioxidant, anti-inflammatory, and anti-apoptotic protective effects on cells and organs, with properties to mitigate various diseases. This section summarizes currently available data regarding the protective role of hydrogen in medicine.

Carbon-13 (^13^C) represents the most stable isotope from a practical point of view. ^13^C-breath tests have recently been developed as a non-radioactive alternative in the clinical setting. In particular, the ^13^C-labeled urea breath test has been widely employed clinically to monitor *Helicobacter pylori *infections. In section 6, two carbon-13 research experts provide the chapter entitled "Gas and Medical Application: V. ^13^C" (pages 100-117). They also discuss carbon-13's advantages and disadvantages.

Further chapters describe other gases and medical application in section seven, "Gas and Medical Application: VI. Others" (pages 119-143) with useful additions. Dr. Sasaki *et al. *report very interesting results in a chapter entitled "Acetone Response during Graded and Prolonged Exercise" (pages 119-124). In expired air, acetone, one of the ketone bodies, gradually increases during graded and prolonged exercise in experiments with healthy human volunteers. Breath acetone levels correlate with fat oxidation rates during exercise. Dr. Tsuda *et al. *discuss a very interesting topic, "Findings of Skin Gases and Their Possibilities in Healthcare Monitoring" (pages 125-132). Skin gas, which is emanated from human skin, is considered to be another option for noninvasive clinical monitoring in human healthcare, as shown in Table 1 (page 126). The skin gases include acetone, ethanol, hydrogen, ammonia, methane, nitrogen monoxide, and carbon monoxide. The concentration of the components in skin gas is closely related to that in blood, and in some cases, in breath. Lastly, phytoncides, antimicrobial allelochemic volatile organic compounds derived from plants, are covered by Dr. Nomura in the chapter entitled "Phytoncide-Its Properties and Applications in Practical Use" (pages 133-143). Phytoncides are used in Inhalation of phytoncides restrains activation of the circular and sympathetic nervous systems, thereby reducing stress and reforming mental function, shown in "forest therapy," as well as in "aroma therapy".

In conclusion, *Gas Biology Research in Clinical Research *is a fascinating and engrossing book on an important topic. This well-written book contains contemporary information and is organized into a clear and readable format. Abundant and appropriate references appear at the end of each chapter. When looking through the table of contents, I realized that this is one of those rare books that suits all levels of readers, from beginner to advanced. Medical gases may have a huge impact as a novel and innovative therapeutic tool for unmet medical needs with considerable health burdens. Although seemingly geared to gas biology scientists, this book should be read by everyone, especially those who work in clinical settings. Drs. Naito and Yoshikawa have certainly fulfilled their goals in editing and writing this book.

## Competing interests

The authors declare that they have no competing interests.

## Authors' contributions

AN and YT conceived of the study, and drafted the manuscript. All authors read and approved the final manuscript.

